# Elongator promotes neuritogenesis *via* regulation of tau stability through acly activity

**DOI:** 10.3389/fcell.2022.1015125

**Published:** 2022-10-26

**Authors:** Michal Shilian, Aviel Even, Hila Gast, Laurent Nguyen, Miguel Weil

**Affiliations:** ^1^ Laboratory for Neurodegenerative Diseases and Personalized Medicine, The Shmunis School of Biomedicine and Cancer Research, The George S. Wise Faculty for Life Sciences, Sagol School of Neurosciences, Tel Aviv University, Tel Aviv, Israel; ^2^ GIGA-Stem Cells and GIGA-Neurosciences, Interdisciplinary Cluster for Applied Genoproteomics (GIGAR), University of Liège, C.H.U. Sart Tilman, Belgium, BIOMED Research Institute, Hasselt, Belgium

**Keywords:** elongator complex, MAPT/Tau protein, neuritogenesis, protein acetylation, familial dysautonomia

## Abstract

The six subunits (Elp1 to Elp6) Elongator complex promotes specific uridine modifications in tRNA’s wobble site. Moreover, this complex has been indirectly involved in the regulation of α-tubulin acetylation in microtubules (MTs) *via* the stabilization of ATP-Citrate Lyase (Acly), the main cytosolic source of acetyl-CoA production in cells, a key substrate used for global protein acetylation. Here, we report additional evidence that Elongator activity is important for proper cytoskeleton remodeling as cells lacking expression of Elp1 show morphology impairment; including distinct neurite process formation and disorganization and instability of MTs. Here, we show that loss of Elongator results in a reduction of expression of the microtubule associated protein Tau (MAPT). Tau, is a well-known key MT regulator in neurons whose lysines can be competitively acetylated or ubiquitylated. Therefore, we tested whether Tau is an indirect acetylation target of Elongator. We found that a reduction of Elongator activity leads to a decrease of lysine acetylation on Tau that favors its proteasomal degradation. This phenotype was prevented by using selective deacetylase or proteasomal inhibitors. Moreover, our data demonstrate that Acly’s activity regulates the mechanism underlying Tau mediated neurite morphology defects found in Elp1 KD since both Tau levels and neurites morphology are restored due to Acly overexpression. This suggests a possible involvement of both Tau and Acly dysfunction in Familial Dysautonomia (FD), which is an autosomal recessive peripheral neuropathy caused by mutation in the *ELP1* gene that severely affects Elp1 expression levels in the nervous system in FD patients in a similar way as found previously in Elp1 KD neuroblastoma cells.

## Introduction

The best established cellular function of the Elongator complex is translation through modification of uridines in tRNA’s wobble site. The Elongator complex promotes the formation of 5-methoxycarbonylmethyl (mcm5) and 5-carbamoylmethyl (ncm5) on side-chains of wobble uridines (U34) of selected tRNAs, thereby regulating protein translation. Reduced levels of modified anticodons caused by depletion of the Elongator complex results in a codon-dependent decrease in ribosomal translocation and affect protein synthesis rates globally (Huang, Johansson and Byström, 2005; Hermand, 2020). Moreover, this complex has a role in intracellular transport in the nervous system (Singh et al., 2010; Miśkiewicz et al., 2011) through acetylation of the α-tubulin in neuronal microtubules (MT) (Creppe et al., 2009; Solinger et al., 2010; Even et al., 2019; Even et al., 2021). Loss of Elongator activity is associated with both neurodegeneration and axonal transport defects (Solinger et al., 2010; Bento-Abreu et al., 2018) *in vivo*, and also leads to Familial Dysautonomia (FD). FD is an autosomal recessive neuropathy that affects the development and function of the autonomic and peripheral nervous system (PNS) (Riley and Day, 1949; Bar-Aluma, 2003). A point mutation in the gene coding for the Elongator scaffold subunit, *ELP1*, results in its splicing defects *via* exon skipping, further leading to a severe reduction of Elp1 protein levels in the FD patient nervous system (Anderson et al., 2001; Slaugenhaupt et al., 2001). Elongator is a complex made of two copies of six subunits (Elp1 to Elp6) where Elp3 acts as the catalytic ones (Winkler et al., 2002; Petrakis, Wittschieben and Svejstrup, 2004). Loss of Elp1 results in impaired Elongator activities, and removal of any Elongator subunits affects the Elongator assembly leading to comparable phenotype in eukaryotes, suggesting that all subunits are essential for the integrity and activity of the complex (Petrakis, Wittschieben and Svejstrup, 2004; Close et al., 2006; Esberg et al., 2006). Our FD neuroblastoma cell model, where Elp1 is knockdown, shows transcriptional changes and aberrant cell shape, expressed as cell adhesion problem that results from Contactin 1 overexpression, including distinct neurite process formation as well as disorganization and instability of microtubules (MTs) (Cohen-kupiec et al., 2010; Cheishvili et al., 2011; Cohen-Kupiec et al., 2011).

The nervous system homeostasis depends on the integrity, dynamics, and organization of MT cytoskeleton (Hirokawa et al., 1996), which is regulated by various microtubule associated proteins (MAPs). The key regulator MAP Tau (MAPT) is associated with MTs and promotes their assembly and stabilization in neurons. Tau likely plays a key role in axonal growth and in the establishment of neuronal polarity and strongly promotes neurite outgrowth during differentiation (Caceres, Potrebic and Kosik, 1991; Esmaeli-azad, Mccarty and Feinstein, 1994; Hirokawa et al., 1996). In contrast, Tau malfunction underlies neurodegeneration and is associated with frontotemporal dementia, Alzheimer, Parkinson and other Tauopathies (Froelich et al., 1998; Spillantini et al., 1998; Ludolph et al., 2009). Tau proteins undergo a large variety of posttranslational modifications (PTMs) that influence it structure and function (Martin, Latypova and Terro, 2011; Mietelska-Porowska et al., 2014). For example, Tau acetylation mediated by the acetyltransferase p300 and its deacetylation by Hdac6 regulates its turnover and stability (Min et al., 2010; Cook and Stankowski, 2014). Ubiquitination is another PTM that modulates turnover. Tau ubiquitination mediates its degradation in the cytosol by the ubiquitin proteasome system (UPS) (David et al., 2002). Interestingly, nearly all acetylation sites on Tau are alternately modified by ubiquitin (Min et al., 2010), and evidence from primary cultured neurons and *in vivo* experiments suggested that competition between acetylation and ubiquitylation of lysine sites in Tau (Min et al., 2010) takes place, that further affect its protein turnover.

We recently showed that loss of Elongator activity results in lower MT acetylation in projection neurons, across species. This phenotype results from a loss of stabilization of ATP-Citrate Lyase (Acly), thereby affecting the acetylase activity of alpha-Tat1 and ultimately resulting in abnormal vesicular transport in projection neurons *in vivo* and *in vitro* (Even et al., 2021). Acly is a transferase that catalyzes the conversion of citrate and coenzyme A to acetyl-CoA, which is one key substrate used for protein acetylation. It is thus possible that loss of Elongator indirectly impacts the activity of multiple acetyltransferases that mediated PTM processes in proteins of both the cytosol and the nucleus, as we have reported for α-tubulin in MTs (Even et al., 2021) and H3 histones by others (Martin et al., 2011) (Wellen et al., 2009), respectively.

Here, by combining cellular and molecular analyses in a neuroblastoma cell line depleted for Elp1 and also in several Elp3 depletion models *in vitro* and *in vivo* we show that loss of Elongator results in reduced Tau protein levels. This results in the abnormal morphology of the neurite’s network. At the molecular level, the reduction of Tau level results from its increased instability that occurs upon changes in the balance between ubiquitylation and acetylation. The reduction of Elongator activity leads to a decrease of lysine acetylation on Tau protein that favors its proteasomal degradation. This results from Acly decreased activity under Elongator deficiency in these cells. Moreover, our data demonstrate that Acly’s activity regulates the mechanism underlying Tau mediated neurite morphology defects found in Elp1 KD suggesting the possible involvement of Tau dysfunction in the FD peripheral neuropathy.

## Materials and methods

### Cell culture

SH-SY5Y human neuroblastoma-derived cell lines used in this work were cultured in polystyrene culture flasks (Corning) at 37°C with 5% CO2 in DMEM (Gibco, Invitrogen, 11965092) medium supplemented with 10% Fetal Calf Serum (Gibco, Invitrogen, 10500056), 1 mM Sodium pyruvate (Gibco, Invitrogen, 11360070) and antibiotics (50 U/ml of penicillin, streptomycin and nystatin).

HEK293 cells were grown in the same conditions as above. The SHSY5Y human neuroblastoma FD model (Elp1 KD) and PLKO vector control were generated in our laboratory as previously described (Cohen-kupiec et al., 2010). The SHSY5Y human neuroblastoma Tau knockdown model (Tau KD) and control were kindly provided by Dr. Paganetti’s Laboratory for Biomedical Neurosciences (LBN) Neurocenter of Southern Switzerland (NSI) (Sola et al., 2020).

### Neuronal differentiation of neuroblastoma cells

For neuronal differentiation of neuroblastoma cell lines, 96 well tissue culture (microscopy grade) plates were pre-coated with 10 µg/ml Poly-D-Lysine (30–70 kDa, Sigma-Aldrich Corp., Israel) for 4 h at room temperature, then rinsed twice with PBS and incubated with 4 µg/ml Laminin (Sigma-Aldrich Corp., Israel) overnight at 4°C. The plates were rinsed once with PBS and neuroblastoma cells were seeded in full supplemented DMEM, after 24 h the cells were incubated with 10 µM retinoic acid for 5 days and then in serum-free medium (Gibco, Invitrogen, 11965092) with 2 nM BDNF (PeproTech Asia, Israel) for 3 days at 37°C with 5% CO2.

### Animals

Brains from Elp3cKO and WT mice at P0-P2 were collected and snap frozen by the Laboratory of Molecular Regulation of Neurogenesis, University of Liège, Belgium and sent frozen to the Laboratory in Israel for analysis (Even et al., 2021). *Drosophila melanogaster* fly stocks were obtained from the Laboratory of Molecular Regulation of Neurogenesis, University of Liège, Belgium (Even et al., 2021). Flies were kept at 25°C in incubator with regular 12 h light and dark cycle. All crosses were performed at 25°C. UAS-RNAi carrying lines; UAS:RNAi Zpg (VDRC CG10125), UAS:RNAi Elp3 (VDRC CG15433) were crossed with Elav-Gal4 (BDSC 458) for WB analysis.

### Image based cell HCA live phenotyping experiments

For live HCA microscopy experiments, 8,000 neuroblastoma cells were plated per well in 96-well plates (Grenier, Austria) in full supplemented DMEM and incubated overnight at 37°C, and 5% CO2 before starting neuronal differentiation with RA as described above. For live staining, culture media was removed and replaced with fluorescent dyes mix containing Hoechst 33342 (Merck-Sigma, United States) 1:10,000, CellTrace™ Calcein Green AM (Invitrogen, United States) 1:5,000 or Calcein Red-Orange AM (Invitrogen, United States) 1:5,000, diluted in HBSS. After 30 min incubation at 37°C, and 5% CO2 the plate was transferred to the IN Cell Analyzer 2,200 (GE Healthcare) for image acquisition under cell culture environmental conditions. Twenty images per fluorescent channel (fixed spacing fields) for each well were acquired in two different channels in less than 60 min. All images under a ×20 magnification were taken using the same acquisition protocol with constant exposures for each fluorescent channel. The multiple cell images were subsequently segmented and high content analyzed using IN Carta^®^ Image Analysis Software. Two dimension principal component analysis (PCA) was applied based on morphological features, followed by a ranking of features contributing to the separation in the PCA analysis.

### Immunofluorescence

Cells were plated on coverslips in 24 well plates at a density of 30,000 cells following neuronal differentiation (as described above). Cells were fixed by incubation in 4% PFA-sucrose in PBS for 10 min at RT and washed twice with PBS. Permeabilization was done using PBS + 0.1% Triton X for 10 min. Cells were incubated in blocking solution (PBS+0.05% Triton-X+10% FBS+ 2% BSA) for 1 h at RT. Following overnight incubation with primary antibodies (see Table 1) in blocking solution at 4°C, washing, and incubation with secondary antibodies (PBS+0.05% Triton-X+10% FBS+ 2% BSA) at RT for 1 h, nuclei were stained for 10 min with 1:10,000 Hoechst 33342/PBS and coverslips were washed and then mounted on microscope slides. Images of the stained cells were obtained using a confocal microscope Zeiss LSM 510 Meta. Fluorescence intensity levels of Tau labeled cells were measured by Fiji (https://imagej.net/Fiji/Downloads).

**TABLE 1:** List of Antibodies.

Protein	Company	Cat #	WB	IF	IP
Tau HT7	Invitrogen	MN1000B	1:1000	1:400	5ug
Tau	Abcam	ab64193	1:500		
Tau 5A6	Hybridoma Bank		1:500		
Elp1/IKAP	Abnova	PAB12857	1:1000		
Elp3	Jesper Svejstrup		1:1,000		
β-actin	Sigma-Aldrich	A3854	1:20,000		
α-Tubulin	Sigma-Aldrich	T9026	1:5,000		
Acetylated α-Tubulin	Sigma-Aldrich	T7451	1:15,000		
Acly	Cell Signaling	13390	1:1,000		
Hdac6	Santa Cruz Biotechnology	sc-5258	1:200		
p300	Cell Signaling	D8Z4E	1:1000		
Ac-Lysine	Sigma-Aldrich	SAB5200090	1:200		
Ubiquitin	Sigma-Aldrich	u0508	1:1000		
HA	Sigma	H6908			
HRP-conjugated donkey anti-rabbit	Abcam	ab 205722	1:10,000		
HRP-conjugated donkey anti-mouse	Abcam	ab97040	1:10,000		
goat anti mouse	Life technologies	A-10680		1:400	
goat anti rabbit	Life technologies	A-21244		1:400	

### Real time quantitative PCR analysis

Total RNA was extracted with TRIzol Reagent (Ambion, Life Technologies) followed by RNA extraction performed following the manufacturer’s instructions. After DNAse treatment (Roche), The concentration of total RNA was measured using a Nano Drop Spectrophotometer (Nano Drop Technologies, United States). Total RNA (300 ng) was reverse-transcribed into complementary DNA (cDNA) with Reverse It first Strand kit (ABgene) using oligo-dT as a primer according to the manufacturer’s instructions.

RT-qPCR was performed using Quant Studio (Thermo) and TaqMan primers, HPRT gene Hs99999909_m1, was used as endogenous gene control. All quantitative real-time PCR TaqMan analysis are presented as representative results in triplicates of 3 biological repeats.

Analyses were done using the 2-^ΔΔ^CT method with the following primers; for *ELP1* Hs00932050_m1 and *MAPT* Hs00902194_m1.

### Western blot

Cells were quickly homogenized on ice in RIPA buffer, protease inhibitor cocktail (Sigma-Aldrich, S8820) and 5 μM Trichostatin-A (TSA, Sigma-Aldrich, T8552) were added to the buffer to inhibit protein degradation and deacetylation. Protein concentration of each sample was measured by using the Protein Assay Kit (Pierce Biotechnology, Rockford) according to the manufacturer’s instructions. Subsequently, samples were denatured by 10 min incubation in 70°C in loading buffer and reducing agent (life technology), and were loaded on SDS-page gel 4–12% gradient gels (life technology) in MES SDS running buffer (life technology) to be finally transferred to a nitrocellulose membrane in 7 min using an iBlot-2. Immunoblotting was performed with the primary and secondary antibodies listed in Table 1. We used 2 μg of protein lysate for α-tubulin acetylation analysis and 20–30 μg for all other proteins. Nitrocellulose membranes were imaged using Amersham Imager 600 (General Electric, 29083461) and band densitometry was measured using FIJI.

### Cycloheximide pulse-chase assay

Differentiated neuroblastoma Elp1 KD and control cells were cultured in 6-well plates at 80% confluency before treatment with 50 μg/ml cycloheximide (CHX) (C7698 Sigma) for 0 h, 2 h, 8 h, 10 h, 24 h. All samples were harvested simultaneously by adding in each well 100 μL RIPA buffer (supplemented with complete proteinase inhibitor cocktail and TSA) and subsequent mechanical scraping. Following incubation on ice for 10 min, samples were centrifuged for 10 min at 8,000 x g to collect the supernatant. Protein concentration of each sample was measured by using the Protein Assay Kit (Pierce Biotechnology, Rockford) according to the manufacturer’s instructions. following WB analysis (as described above).

### Reagents and drug treatments

BMS 303141 (BMS), Tubastatin A (TubA), MG-132 and cycloheximide were obtained from Sigma-Aldrich (SML0784, SML0044, M7449, and C104450, respectively). MG-132 (20 µM) was added to fully differentiated cells 4 h prior to protein extraction. For ubiquitinated Tau MG-132 (17.5 µM) was added to HEK293 transfected cells 15 h prior to immunoprecipitation. Treatment of TubA, (2 µM) for 24 h alone or together with MG-132 (20 µM) for the last 4 h were added to fully differentiated cells, prior to protein extraction. BMS (1 µM) was added to fully differentiated cells for 72 h prior to cells fixation and immunofluorescence analysis.

### Acly activity assay

Acly activity assay was measured as previously described by us (Even et al., 2021) using 5 μg of protein extracts from differentiated neuroblastoma Elp1 KD and control cells.

### Plasmids and transfections

HEK293 cells were transfected with 5–10 μg of plasmids pCAGGs hElp3 Flag IRES RFP, pCAGGs hElp3mKAT Flag IRES RFP (Laguesse et al., 2015), using calcium phosphate. 24 h post transfection, cells were lysed in IP lysis buffer on ice, following immunoprecipitation (as described below).

SH-SY5Y human neuroblastoma cells were infected using Adenoviruses MOI 1000 for 4 h; The Adenovirus vector is VB211014, pAV [Exp]-EGFP-EFS > hACLY [NM_001303274.1]:IRES:Neo for expression of human ACLY or pAV [Exp]-EGFP-EFS>hMAPT [NM_001377265.1]:IRES:Neo for expression of human MAPT, and the promotor was replaced by an EFS promoter. After 24 h cells were harvested and plated on PDL-Laminin coated 96 wells plate, 24 h later a shorter neuronal differentiation protocol (3 days RA and 3 days BDNF) was applied and image based cell HCA live phenotyping experiment was performed.

### Immunoprecipitation analysis

Pierce Crosslink immunoprecipitation kit (#26147) was used following the manufacturer’s instructions. For immunoprecipitation experiments, cells were lysed in IP buffer (supplied with the kit) and supplemented with protease inhibitors and TSA. About 300–1000 μg of cell lysates were incubated overnight at 4°C with the indicated antibodies. For Tau acetylation and ubiquitylation analysis, 5 μg of Tau HT7 (Invitrogen) were crosslinked using DSS to protein A/G. For detection by WB of acetylated Tau or ubiquitinated Tau, acetylated Lysine (Sigma) antibody or ubiquitin (Sigma) or HA (Sigma) and Tau (Abcam) or Tau HT7 (Invitrogen) were used. acetylated Tau or ubiquitinated Tau were measured as ac-Lys levels or ubiquitin levels divided by Tau levels.

### Deacetylation assay

α-Tubulin deacetylation assay was performed using 10 μg of protein extracts from differentiated SHSY5Y neuroblastoma cells as previously described by us (Even et al., 2021).

### *In vitro* α-tubulin assay

*In vitro* α-tubulin acetylation assay was performed using 25 μg of protein extracts from differentiated SHSY5Y neuroblastoma cells as previously described by us (Even et al., 2021).

### Acetyl-CoA sample preparation and LC–MS/MS analysis

Acetyl-CoA sample preparation and LC–MS/MS analysis was performed as previously described by us (Even et al., 2021).

### Analysis and statistics

All experiments were performed under single blinded conditions and statistical analyses were generated with GraphPad Prism Software 7.0.

## Results

**FIGURE 1:**
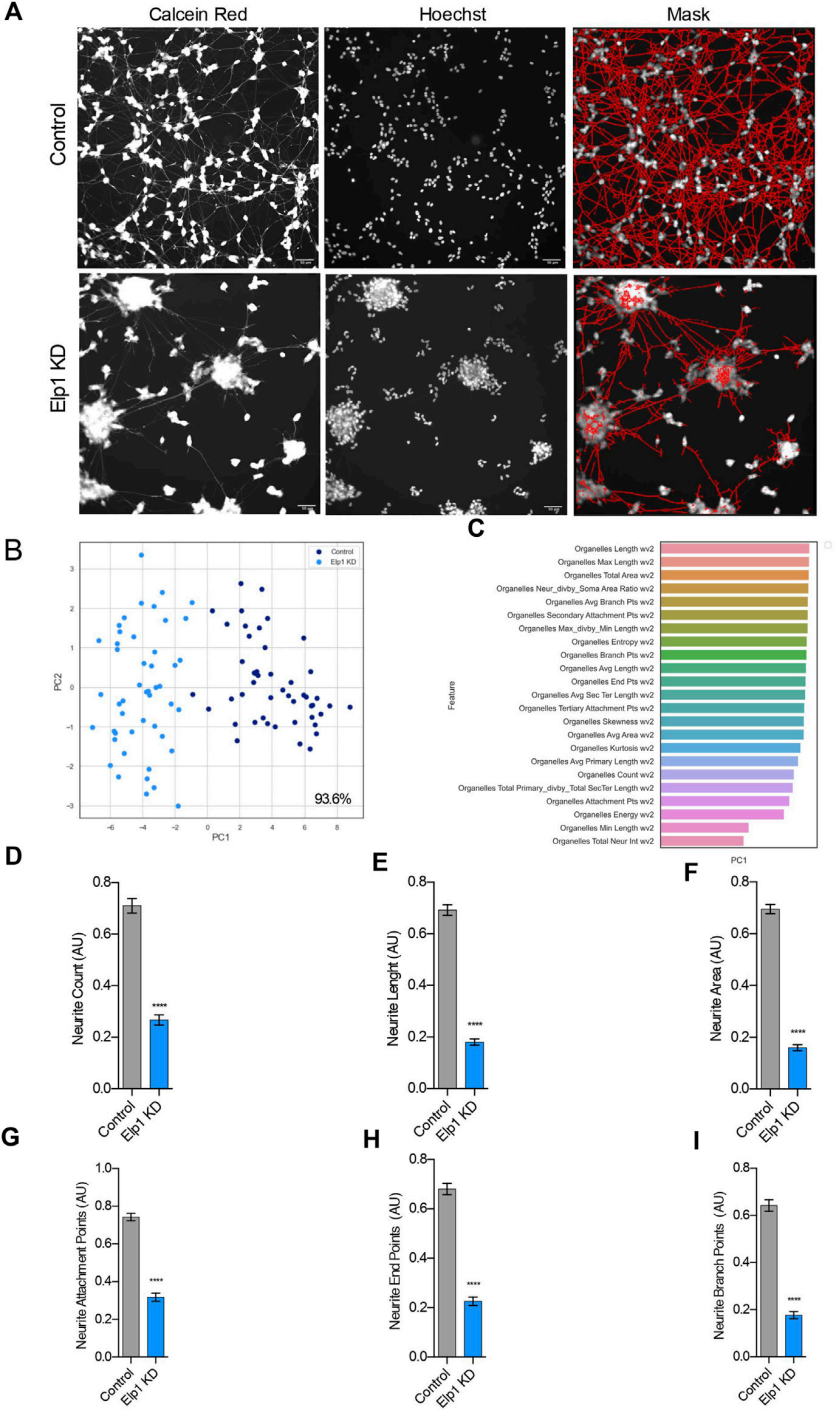
Elp1 is involved in neurite morphology, Depletion of Elp1 Causes Defective neurite outgrowth in differentiated SHSY5Y cells. **(A)** Representative images of Live fluorescence microscopy staining of differentiated SHSY5Y cells control *versus* Elp1 KD using Calcein AM (Red) and Hoechst (Blue) to visualize neurite morphology. on the right side; segmentation of neurites by InCarta software. Scale bar is 50 µm. **(B)** Two dimension principal component analysis (PCA) plot of stained cells based on morphological features. Symbols in dark blue represent the control cells; symbols in light blue represent Elp1KD cells. each circle represent data extracted from 20 fields coming from one well, each field contain hundreds of cells. PCA Score 93.6% **(C)** Plot of features contribution to the separation in the PCA analysis **(D–I)** Quantification of the selected morphology features including Neurite Count **(D)**, Neurite Length **(E)**, Neurite Area **(F)**, Neurite Attachment Points **(G)**, Neurite End Points **(H)** and Neurite Branch Points **(I)**. All graphs show values of means ± SEM. Significance was determined by: d-i two-sided *t* test, Specifically, [**(D)** *p* < 0.0001; **(E)** *p* < 0.0001; **(F)** *p* < 0.0001; **(G)** *p* < 0.0001; **(H)** *p* < 0.0001; **(I)** *p* < 0.0001; ] n = Number of wells (each well contains data from 20 fields, each field contains hundreds of cells): **(D–I)** Control n = 48; Elp1 KD *n* = 48.

To investigate further and quantify the morphological differences previously observed between Elp1 KD and control neuroblastoma cells (Cohen-kupiec et al., 2010; Cheishvili et al., 2011) we applied live fluorescence microscopy image high content analysis (HCA) of Calcein AM live stained cells to visualize neurite morphologies. For the morphology experiments the neuroblastoma cells were seeded in 96 well plates and cultured in differentiation medium for 5 days containing 10% FBS medium with retinoic acid (RA), followed by serum-free medium supplemented with BDNF for 3 days. For neurite morphology analysis, we identified neurite processes using a specialized image analysis software in thousands of double stained cells, calcein AM (left panel) and Hoescht 222342 (mid panel), as represented in Figure 1A (right panel) for control and Elp1 KD respectively.

A two-dimensional principal component analysis was applied to identify the vectors from the data produced in these experiments that classify the difference in neurite morphology between control (in blue) and Elp1 KD (in light blue) cells (Figure 1B). Feature selection analysis show the list of critical features (Figure 1C) that best contribute to the classification difference between the groups. We focused in the most meaningful and relevant features that characterize the morphological differences of neurites between control and Elp1 KD for further analysis as shown in Figures 1D–I. All the selected neurites morphology features displayed distinct and significant differences between control and Elp1 KD cells (Figures 1D–I). Overall, the reduced neurite number, length and other morphological features in Elp1 KD cells suggest that Elp1 is required for neuritogenesis and depletion of Elp1 causes defective morphology of neurites. This result is in line with our previous studies where we found that Elp1 is involved in neuronal outgrowth and peripheral target innervation in chick embryos (Abashidze et al., 2014).

Since neurite growth and morphology rely on several cytoskeleton proteins, such as Microtubule associated proteins (MAPs), we further aimed to investigate whether the observed Elp1-dependent neurites morphology differences in FD neuroblastoma model can be explained by deregulation of Tau (Microtubule Associated Protein Tau; MAPT).

**FIGURE 2:**
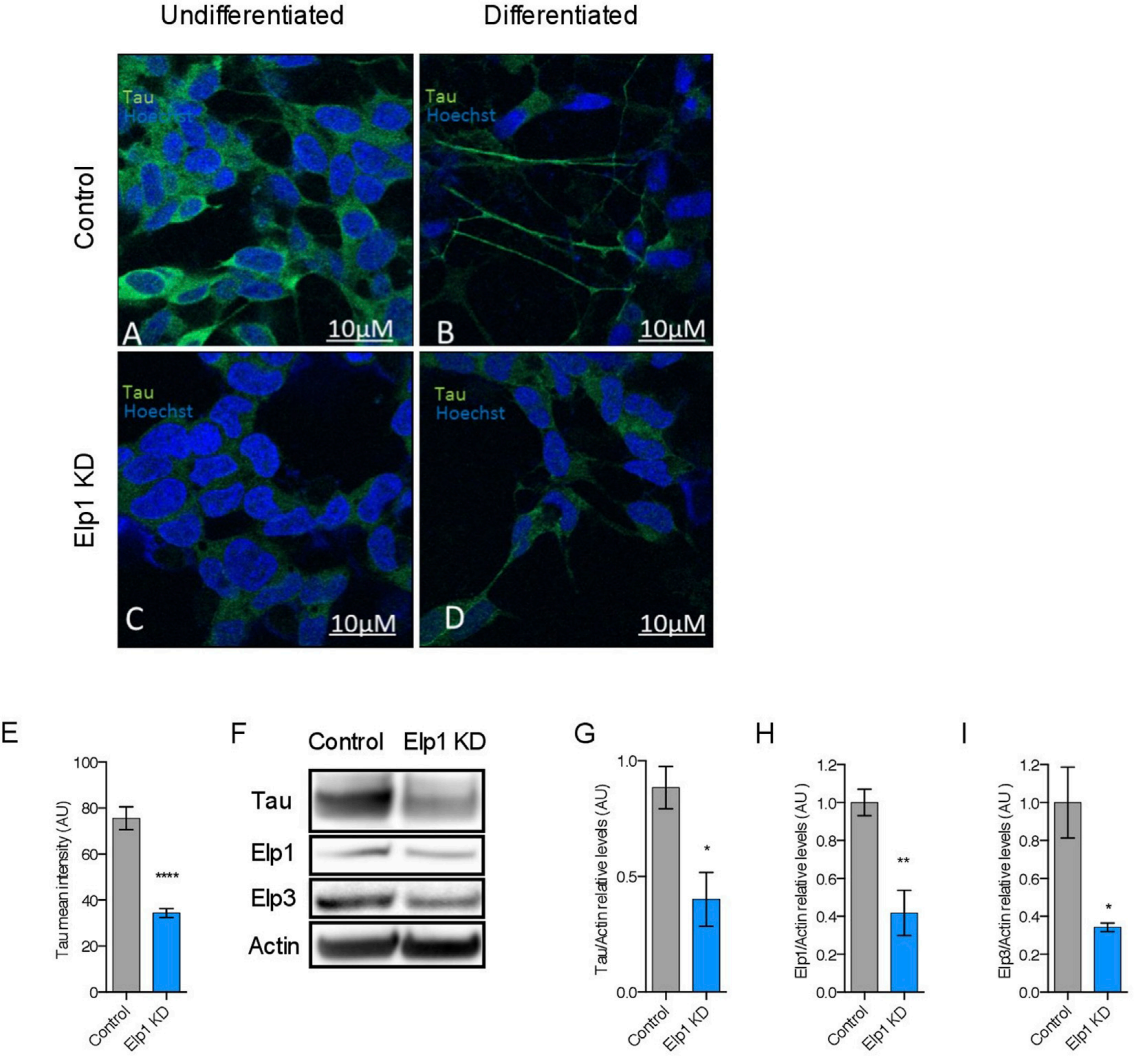
Elongator depletion leads to reduction in Tau protein expression. **(A–E)** Immunolabeling and quantification of Tau in Elp1 KD and control SHSY5Y cells. Tau is stained in green, before **(A,C)** and after differentiation **(B,D)**. Scale bar is 10 µm. **(F–I)** Western blotting to detect and quantify Tau, Elp1, Elp3 and ß-Actin in differentiated SHSY5Y extracts from Control and Elp1 KD cells. Histograms of proportion of Tau **(G)**, Elp1 **(H)** and Elp3 **(I)** expression to ß-Actin. All graphs show values of means ± SEM. Significance was determined by: eg-i two-sided *t* test, Specifically, [**(E)** *p* < 0.0001; **(G)** *p* = 0.0114; **(H)** *p* = 0.0018; **(I)** *p* = 0.0249; ]. *n* = Number of wells (each well contains data from 20 fields, each field contains hundreds of cells): **(E)** Control *n* = 119; Elp1 KD n = 85; *n* = Number of experimental repeats: **(G)** Control *n* = 5; Elp1 KD *n* = 5; **(H)** Control *n* = 6; Elp1 KD *n* = 6; **(I)** Control n = 3; Elp1 KD *n* = 3.

To investigate the correlation between the neurite morphological features with Tau expression in Elp1 KD FD neuroblastoma cells, we measured Tau expression by immuno-fluorescence confocal microscopy analysis of Elp1 KD and control cells, using Tau antibodies before and after differentiation with RA and BDNF, as shown in representative images in Figure 2 A-D. Note the apparent morphological differences that exist in neurite length between control and Elp1 KD, especially after differentiation (Figures 2B,D respectively). Quantitative analysis of Tau fluorescence intensity in differentiated cells (Figure 2E) shows that Tau levels in control neuroblastoma cells both before and after differentiation (Figures 2A,C, respectively) are higher compared to Elp1 KD (Figures 2B,D, respectively). Western blot analysis on protein extracts obtained from differentiated control and Elp1 KD neuroblastoma cells shows a reduction by almost 60% of Tau protein level in Elp1 KD, as compared to control cells (Figures 2F,G). The severe reduction of Tau protein upon Elp1 deficiency is not exclusive to Elp1 KD neuroblastoma cells and is further supported *in vivo* in Elongator deficiency neuronal models in mice and flies (Supplementary Figure S1). Tau protein levels were measured by WB analysis in extracts made of adult mice brains of Elp3 KD (Supplementary Figure S1A, B) and adult fly heads, following neuronal (elav-driven) expression of Elp3 KD (Supplementary FigureS1C, D). These results showed reduced Tau protein levels in both Elp3 KD models, suggesting that loss of Elongator complex impacts Tau expression in human, flies and mouse neuronal models.

**FIGURE 3:**
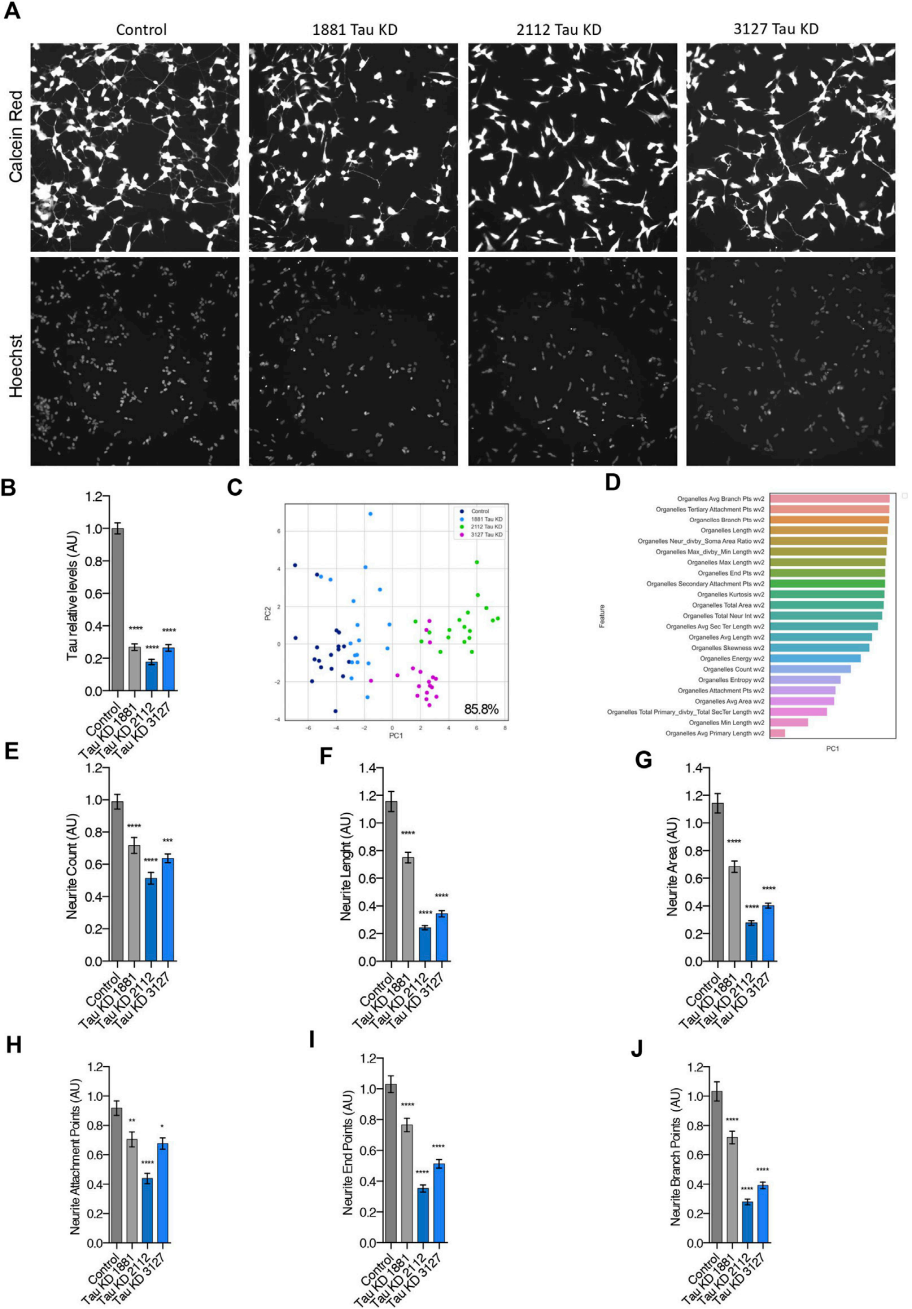
Depletion of Tau causes abnormal neurite morphology in differentiated SHSY5Y cells, similarly as in Elp1 depletion*.* **(A)** Representative images of Live fluorescence microscopy staining of differentiated SHSY5Y cells control *versus* Tau KD lines (Tau KD 1881; Tau KD 2112, Tau KD 3127) using Calcein AM (Red) and Hoechst (Blue) to visualize neurite morphology. Scale bar is 50 µm. **(B)** Immunolabeling and quantification of Tau levels **(C)** Two dimension principal component analysis (PCA) plot of stained cells based on morphological features. Symbols represent the control cells (dark blue); Tau KD 1881 cells (light blue), Tau KD 2112 (pink), Tau KD 3127 (green). each circle represent data extracted from 20 fields coming from one well, each field contain hundreds of cells. PCA Score 85.8% **(D)** Plot of features contribution to the separation in the PCA analysis **(E–J)** Quantification of the selected morphology features including Neurite Count **(E)**, Neurite Length **(F)**, Neurite Area **(G)**, Neurite Attachment Points **(H)**, Neurite End Points **(I)** and Neurite Branch Points **(J)**. All graphs show values of means ± SEM. Significance was determined by: b, e-j two-sided one-way analysis of variance (ANOVA), Specifically, [**(B)** *p* < 0.0001, F = 250.3; **(E)** *p* < 0.0001, F = 23.93; **(F)** *p* < 0.0001, F = 77.28; **(G)** *p* < 0.0001, F = 69.46; **(H)** *p* < 0.0001, F = 22.06; **(I)** *p* < 0.0001, F = 56.87; **(J)** *p* < 0.0001, F = 54.18; ] In addition, the post hoc multiple comparisons, to analyze statistical difference of each condition compared to control for **(B–J)** by Dunnett test, *p < 0.05, **p < 0.01, ***p < 0.001, and ****p < 0.0001. *n* = Number of wells (each well contains data from 20 fields, each field contains hundreds of cells): **(B)** Control n = 40; Tau KD 1881 *n* = 32; Tau KD 2112 *n* = 32; Tau KD 3127 *n* = 24; **(E–J)** Control *n* = 56; Tau KD 1881 *n* = 50; Tau KD 2112 *n* = 62; Tau KD 3127 *n* = 18.

Since previous studies have suggested that Tau may function in neurite outgrowth and growth cone motility and that inactivation of Tau in whole DRG neurons resulted in reduced neurite number and length (Liu, Lee and Jay, 1999), we postulated that the abnormal neurite morphology observed in Elp1 KD neuroblastoma cells might arise from Tau depletion. To directly investigate this hypothesis, we performed live imaging HCA neurites morphological experiments (similar as shown in Figure 1) using three established Tau-knock down (Tau KD) neuroblastoma cells that express constitutively each one of three distinct Tau shRNAs and respective cell line control, obtained as a kind gift from Paganetti’s lab that were generated as described by them (Sola et al., 2020). As shown in Figure 3 the two-dimensional principal component analysis applied to select the critical features from the data produced in these experiments resulted in the same selected critical neurite morphology features from the analysis done for the Elp1KD cells (Figures 3C,D). Measures from the selected morphology features show a reduced number of neurites per cell, reduction in neurite length and area, fewer attachment points, and reduces numbers of branch points and end points per neurite (Figures 3E–J). The reduction levels in all neurite morphology features correlated with Tau expression levels with Tau KD cell lines expressing different levels of Tau protein, with reduced Tau-expression corresponding to 73% for the 1881 shRNA, 82% for the 2112 shRNA and 74% reduced Tau in 3127 shRNA (Figure 3B). Altogether, these results suggest that the aberrant neurite morphology seen upon Elp1 KD may rely on reduced Tau levels.

**FIGURE 4:**
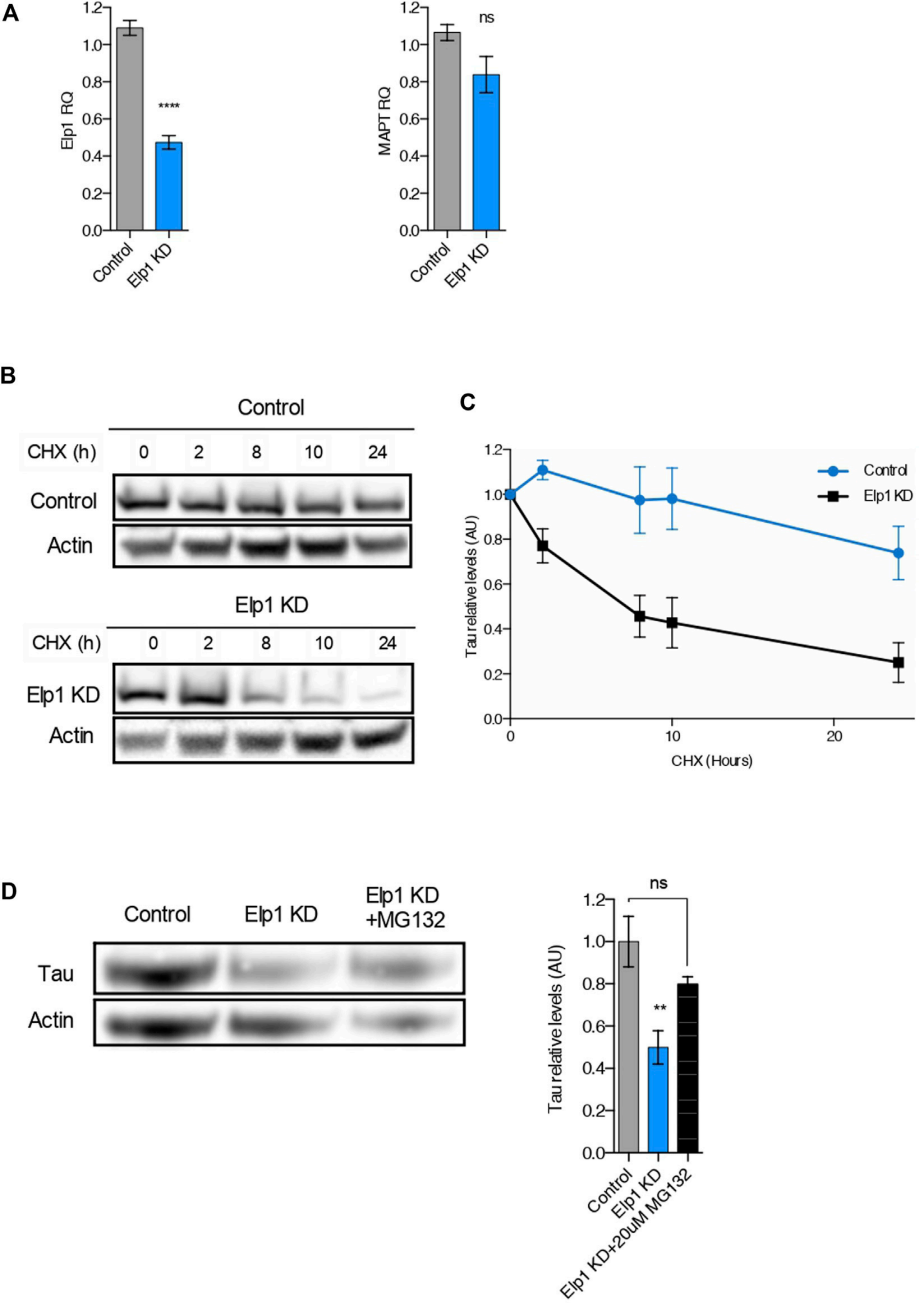
The reduction of Tau levels in ELP1 depletion is due to Tau protein instability*.* **(A)** qRT-PCR analysis of Elp1 mRNA (left) and MAPT mRNA (right) in differentiated SHSY5Y extracts from Control and Elp1 KD cells. **(B,C)** Immunoblotting and quantification of Tau and ß-Actin in differentiated SHSY5Y extracts from Control and Elp1 KD cells incubated with cycloheximide (CHX, 50 μg/ml) for 0, 2, 8, 10 and 24 h. **(D)** Immunoblotting and histogram of Tau and ß-Actin in differentiated SHSY5Y extracts from Control and Elp1 KD cells incubated with vehicle or MG-132 (20 µM) for 4 h. All graphs show values of means ± SEM. Significance was determined by: **(A)** two-sided *t* test, Specifically, [(a left) *p* < 0.0001; (a right) *p* = 0.1349; ] **(C)** two-sided two-way ANOVA. Specifically, [*p* = 0.0224, F Interaction (4, 44) = 3.173] **(D)** two-sided one-way analysis of variance (ANOVA), Specifically, [*p* = 0.0084, F = 8.5; ] In addition, the post hoc multiple comparisons, to analyze statistical difference of each condition compared to control by Dunnett test, **p < 0.01. *n* = Number of experimental repeats: **(A)** Control *n* = 5; Elp1 KD *n* = 5; **(C)** Control *n* = 5; Elp1 KD *n* = 7; **(D)** Control *n* = 4; Elp1 KD *n* = 5; Elp1 KD + MG132 *n* = 3.

It was previously suggested that the Elongator complex contributes to transcriptional elongation *via* RNAPII-associated chromatin remodeling (Close et al., 2006). To test the possibility that the reduction of Tau levels detected in Elp1 knockdown neuroblastoma cells results from a reduction in *MAPT* gene transcription, we performed quantitative real-time PCR analysis (qRT-PCR) as shown in Figure 4A. We observed that while Elp1 mRNA transcription is significantly reduced in Elp1 KD neuroblastoma cells (Figure 4A, left), the MAPT mRNA expression level is similar in both Elp1 KD neuroblastoma cells and control cells supporting the view that Tau expression in the FD model is probably regulated at the protein (Figure 4A, right).

To study if the reduced levels of Tau (see Figure 2) observed in Elp1 KD cells arises from its change of stability, we used the protein synthesis inhibitor cycloheximide (CHX) in pulse-chase experiments, as described in Figures 4B,C. Differentiated neuroblastoma cell lysates were prepared after different times of incubation with CHX and analyzed using primary Tau antibodies by WB analysis (Figure 4B). The turnover rate of Tau in Elp1 KD cells was compared to control cells, with a time of CHX treatment measured as Tau relative levels. Tau showed a faster signal decay in Elp1 KD cells, as compared to control cells, suggesting that Elp1 KD cells have a higher degradation rate of Tau (Figure 4C). To study whether the reduced stability of Tau in Elp1 KD cells correlates to the proteasome-mediated degradation, we examined Tau protein levels in the presence or absence of the proteasomal inhibitor MG132 which blocks the proteasome-mediated degradation (Figure 4D). We found that MG132 increased Tau levels in Elp1 KD cells by almost 2 fold which was similar to levels observed in control cells. Altogether, these results led us to suggest that Elongator may indirectly regulate Tau protein turnover through the ubiquitin proteasome pathway (UPP).

**FIGURE 5:**
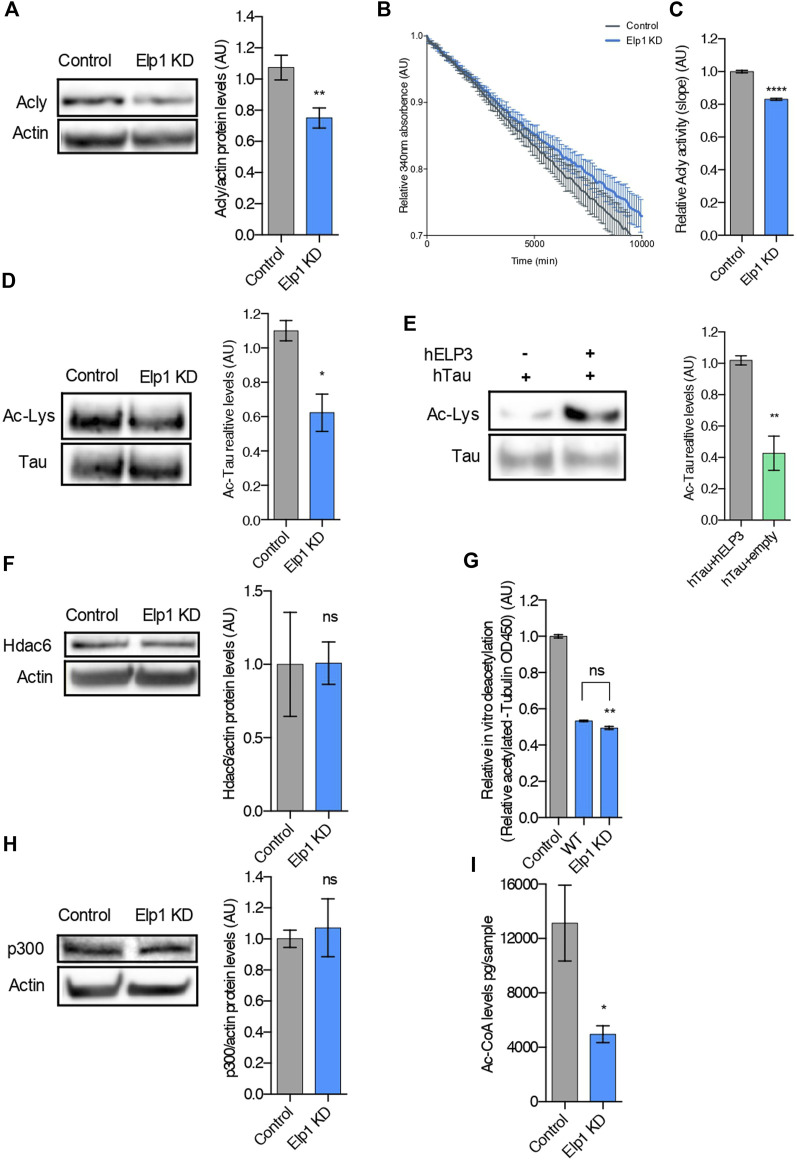
Tau is hypoacetylated, a result of impaired production of acetyl-CoA upon Elongator depletion*.* **(A)** Western blotting to detect and quantify Acly, and ß-Actin in control and Elp1KD cells extracts. Histograms of proportion of Acly expression to ß-Actin. **(B,C)** Analysis of Acly activity by malate dehydrogenase coupled method performed in control and Elp1KD cells lysates. Histogram of relative Acly activity over assay **(B)** and of slopes **(C)** from the linear phase of the reaction. **(D)** Immunoprecipitation followed by Western blotting to detect and quantify Acetylated Tau, in control and Elp1KD cells extracts. Histograms of proportion of acetylated lysine expression to Tau. **(E)** Immunoprecipitation followed by Western blotting to detect and quantify acetylated Tau, in extracts from HEK293 cells transfected with Tau, Elp3 or empty vector. Histograms of proportion of acetylated lysine expression to Tau. **(F)** Western blotting to detect and quantify Hdac6, and ß-Actin in control and Elp1KD cells extracts. Histograms of proportion of Hdac6 expression to ß-Actin. **(G)** *In vitro* deacetylation assay of endogenously acetylated bovine brain tubulin incubated for 4 h with extracts of WT and Elp1KD cells, or without cells extract (control). **(H)** Immunoblotting to detect p300, ß-Actin in control and Elp1KD cells extracts and histograms of proportion of p300 expression to ß-Actin. **(I)** LC-MS quantification of Acetyl-CoA levels in control and Elp1KD cells lysates. All graphs show values of means ± SEM. Significance was determined by: **(A–I)** two-sided *t* test, Specifically, [**(A)** *p* = 0.0059; **(C)** *p* < 0.0001; **(D)** *p* = 0.0256; **(E)** *p* = 0.0019; **(F)** *p* = 0.9833; **(H)** *p* = 0.7225; **(I)** *p* = 0.0456; ] **(G)** two-sided Kruskal Wallis one-way ANOVA, Specifically, [*p* = 0.0003, K = 9.346; ] In addition, the post hoc multiple comparisons, to analyze statistical difference of each condition compared to control by Dunnett test, **p < 0.01. *n* = Number of experimental repeats: **(A)** Control *n* = 8; Elp1 KD *n* = 9; **(C)** Control *n* = 3; Elp1 KD *n* = 3; **(D)** Control *n* = 3; Elp1 KD *n* = 3; **(E)** Control *n* = 4; Elp1 KD *n* = 4; **(F)** Control *n* = 3; Elp1 KD *n* = 3; **(G)** Control *n* = 6; WT *n* = 3; Elp1 KD *n* = 3; **(H)** Control *n* = 4; Elp1 KD *n* = 4; **(I)** Control *n* = 3; Elp1 KD *n* = 3.

Since we recently showed that loss of Elongator activity interferes with MT acetylation and axonal transport *via* reduction of Acly expression (Even et al., 2021), we perform experiments to investigate whether loss of Elp1 may trigger hypoacetylation of Tau (Figure 5). We observed a reduction of Acly protein expression levels also in our Elp1 KD neuroblastoma model (Figure 5A). We next measured Acly activity by performing an *in vitro* acetyl-CoA production assay (malate dehydrogenase coupled assay) (Wellen et al., 2009). For this, we incubated CoA and citrate (Acly substrates) with extracts from Elp1 KD and control neuroblastoma cells. Our results showed that Elp1 KD cells have reduced acetyl-CoA production activity (Figures 5B,C), suggesting that Elp1 depletion affects Acly activity correlated with its decreased expression levels.

It is known that Tau acetylation affects its turnover *via* protein stability (Cook and Stankowski, 2014; Min et al., 2010). To study whether the reduced stability of Tau in Elp1 KD cells results from its impaired acetylation, we measured Tau acetylation levels by immunoprecipitation followed by western blot analysis. For these experiments, we extracted protein from Elp1 KD and control neuroblastoma cells, immunoprecipitated Tau using specific antibodies and revealed by western blot using anti-pan acetyl lysine antibodies. The level of acetylated Tau, measured as ac-lysine levels above total Tau levels, was reduced in Elp1 KD neuroblastoma cells, as compared to control cells (Figure 5D). Next, to test whether Tau acetylation can be increased by Elongator activity, we transiently co-transfected HEK293T cells with Tau and ELP3 or with a control vector. Tau was next immunoprecipitated and subjected to western blot analysis to detect Tau acetylation levels. We observed an increase in acetylated Tau upon its over-expression with Elp3 (Figure 5E). These results suggest that the overall reduction of Tau expression upon reduction of Elongator activity results from the hypoacetylation of Tau.

Since loss of Elongator correlates with poor acetylation of α-tubulin lysine 40 (K40) in neuronal MTs (Creppe et al., 2009; Solinger et al., 2010), a defect shared by Elp1 KD neuroblastoma cells (Supplementary Figure S2A), we tested whether Tau hypoacetylation may arise from a change of expression or activity of Hdac6, the enzyme that also controls α-tubulin deacetylation (Kalebic et al., 2013). Since no change in Hdac6 expression in Elp1 KD cells and control cells (Figure 5F) was observed, we measured the activity of this enzyme in MT preparations. The deacetylation activity of Hdac6 for MTs was assessed *in vitro* by incubating free acetylated α-tubulin (from bovine brain extracts) with extracts from Elp1 KD or control cells (Figure 5G). Together with it, p300 levels, the acetyltransferase which acetylates Tau protein, showed no difference in Elp1 KD cells when compared to control in WB analysis (Figure 5H).

Moreover, we detected a reduction of acetyl-CoA levels in extracts from Elp1 KD cells, as compared to control by LC-MS metabolic analysis (Figure 5I). To assess that the hypoacetylation observed in both Tau and α-tubulin in Elp1 KD neuroblastoma model is caused by lack of acetyl-CoA; the acetyltransferase’s substrate, we conducted an *in vitro* α-tubulin acetylation assay (Even et al., 2019). For this assay, pre-polymerized unacetylated MTs from HeLa cells were incubated with acetyl-CoA and cells extracts from Elp1 KD or control to assess the level of acetylation of α-tubulin. No differences in MT acetylation levels between the Elp1 KD and control cells upon addition of acetyl-CoA were observed (Supplementary Figure S2). Although indirectly, this suggests that Tau hypoacetylation might be a result of reduced Acly and impaired activity, causing a reduction in acetyl-CoA levels in the Elongator depletion background.

**FIGURE 6:**
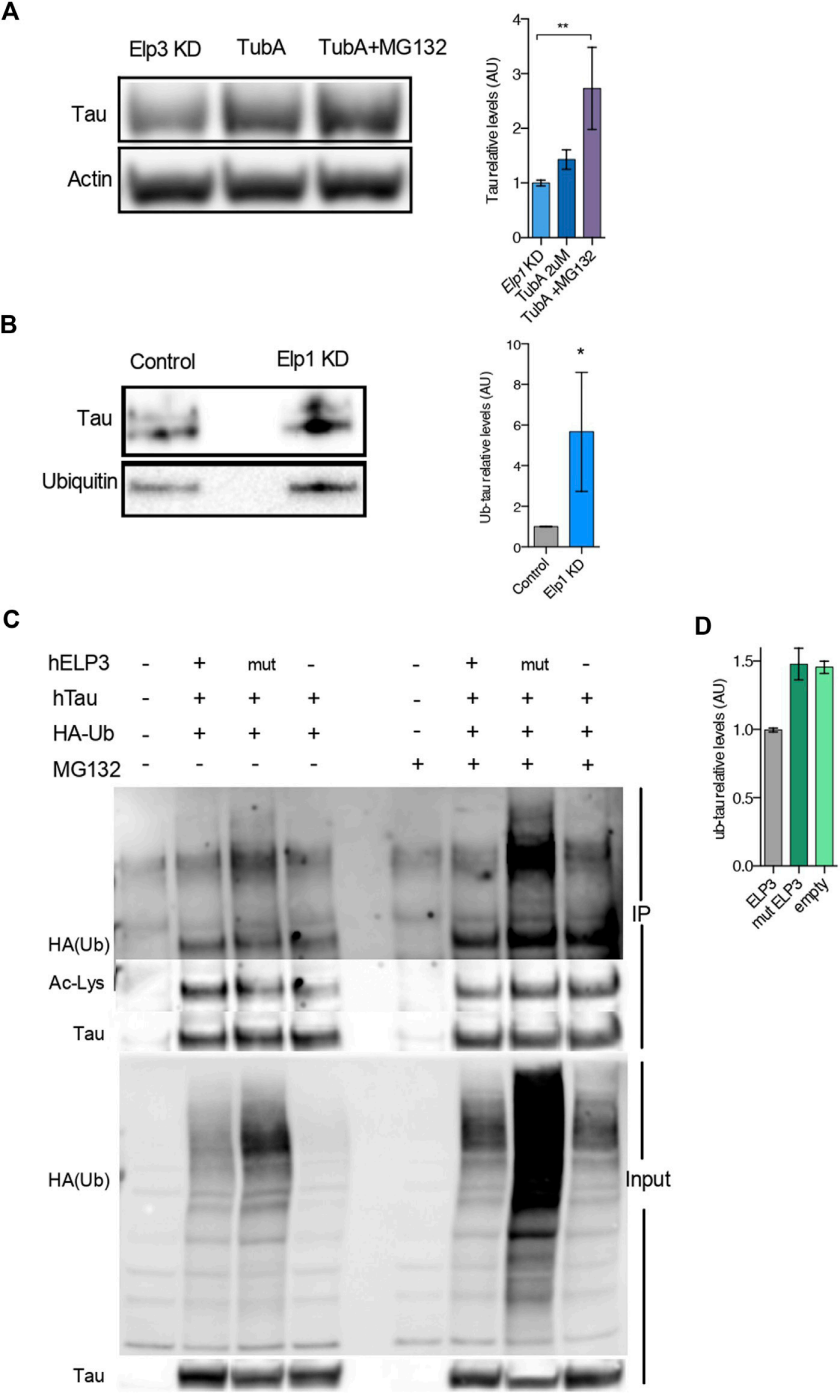
Elongator Deficiency Reduces Tau acetylation, Increases Tau ubiquitylation and induces its Degradation. **(A)** Immunoblotting and quantification of Tau and ß-Actin in differentiated SHSY5Y extracts from Control and Elp1 KD cells incubated with vehicle, Tubastatin A (TubA, 2 µM) for 24 h or Tubastatin A (TubA, 2 µM) for 24 h together with MG-132 (MG132 , 20 µM) for the last 4 h. **(B)** Immunoprecipitation followed by Western blotting to detect and quantify ubiquitylated Tau, in control and Elp1KD cells extracts. Histograms of proportion of ubiquitin expression to Tau. **(C–D)** Immunoprecipitation of Tau followed by Western blotting to detect and quantify ubiquitylated Tau; Lysates from HEK293 cells transfected with plasmids expressing WT Tau, HA-tagged ubiquitin and ELP3 or mutated ELP3 in the KAT domain or empty vector incubated with vehicle or MG-132 (MG132 , 17.5 µM) for 15 h. Immunoprecipitates (IP) and total soluble lysates (Input) were analyzed by western blotting, with either anti-HA or anti-acetylated lysine and anti Tau antibodies. **(D)** Histograms of proportion of ubiquitin expression to Tau incubated with MG-132 (edit graph add stat maybe quantify HA/Tau). All graphs show values of means ± SEM. Significance was determined by: **(A)** two-sided Kruskal Wallis one-way ANOVA, specifically, [*p* = 0.0001, K = 11.8; ] In addition, the post hoc multiple comparisons, to analyze statistical difference of each condition compared to Elp1KD by Dunnett test, *p < 0.05, **p < 0.01. **(B)** two-sided *t* test, Specifically, [**(A)** *p* = 0.0286; ]; *n* = Number of experimental repeats: **(A)** Elp1 KD *n* = 6; Elp1 KD + TubA *n* = 6; Elp1 KD + TubA MG132 *n* = 5; **(B)** Control *n* = 4; Elp1 KD *n* = 4.

To further test whether the reduced level of Tau expression under Elongator depletion background relies on a change in Tau acetylation status and protein degradation, we tested if deacetylation inhibition of Hdac6, which is the known deacetylase of Tau (Noack, Leyk and Richter-Landsberg, 2014), may rescue Tau protein levels in Elongator depleted backgrounds (see Figure 6). To this hypothesis, we measured Tau acetylation in protein extracts from neuroblastoma cells treated with the selective Hdac6 inhibitor Tubastatin A (TubA), combined or not with the proteasomal inhibitor MG-132 treatment. We observed a synergistic increase of Tau level in Elp1 KD cells, as compared to their controls, upon co-inhibition of Hdac6 and proteasomal degradation (Figure 6A). This observation suggests that the stability of Tau is regulated by both UPP and acetylation.

To investigate whether Tau protein turnover is regulated by the UPP and whether Tau ubiquitin levels are affected in Elp1 KD cells, we performed a Western Blot analysis of immunoprecipitated Tau with anti-ubiquitin antibody. Our results show that the ubiquitination levels of Tau are significantly increased in the Elp1 KD cells (Figure 6B). Altogether, these results suggest that the reduction of Tau levels upon Elongator depletion is related to its hypoacetylation and increased ubiquitylation. According to that acetylation and ubiquitination of Tau potentially may occur on similar lysine residues, we hypothesized that Elongator depletion changes the balance of acetylation/ubiquitylation on some lysine residues.

To test this hypothesis, we co-transfected HEK293T cells with HA-tagged ubiquitin, and Tau together with either WT ELP3 (as the Elongator’s functional catalytic subunit) or KAT domain mutant ELP3 or with empty vector, in the presence or absence of the proteasomal inhibitor MG132. We then performed an immunoprecipitation assay with Tau antibodies followed by immunoblotting using an anti-HA antibody and Anti-acetylated lysine antibody, to detect acetylation and ubiquitination of Tau in the same samples. We observed that the acetylation levels of Tau were reduced in mutant ELP3 overexpression and an empty vector control as compared to ELP3 overexpression. Our data showed a characteristic high molecular weight smear of polyubiquitin molecules conjugated to Tau in mutated ELP3 co-transfected cells. In contrast, we detected a reduced ubiquitin signal in cells transfected with ELP3 (Figure 6C HA-ub high molecular weight smear, lanes 2–4). In the presence of MG132, we observed that Tau polyubiquitination levels increased in mutant ELP3 expressing cells as well as with the empty vector when compared to control ELP3 expressing cells (Figure 6C; HA-ub single band, lanes 6–8 and 6E). The results showed that Tau is associated with polyubiquitin chains and that defects in Elp3 led to increased levels of Tau ubiquitylation at the expense of its acetylation (Figure 6D; ac-Lys single band, lanes 2–4). These results support the view that Elp3 is required for proper Tau acetylation and that mutant Elp3 impaired Tau acetylation, thereby disrupting the balance of acetylation/ubiquitylation of Tau lysine residues. These results suggest that the instability of Tau in cells depleted for Elongator activity results from changes in its post translational modifications leading to its degradation.

**FIGURE 7:**
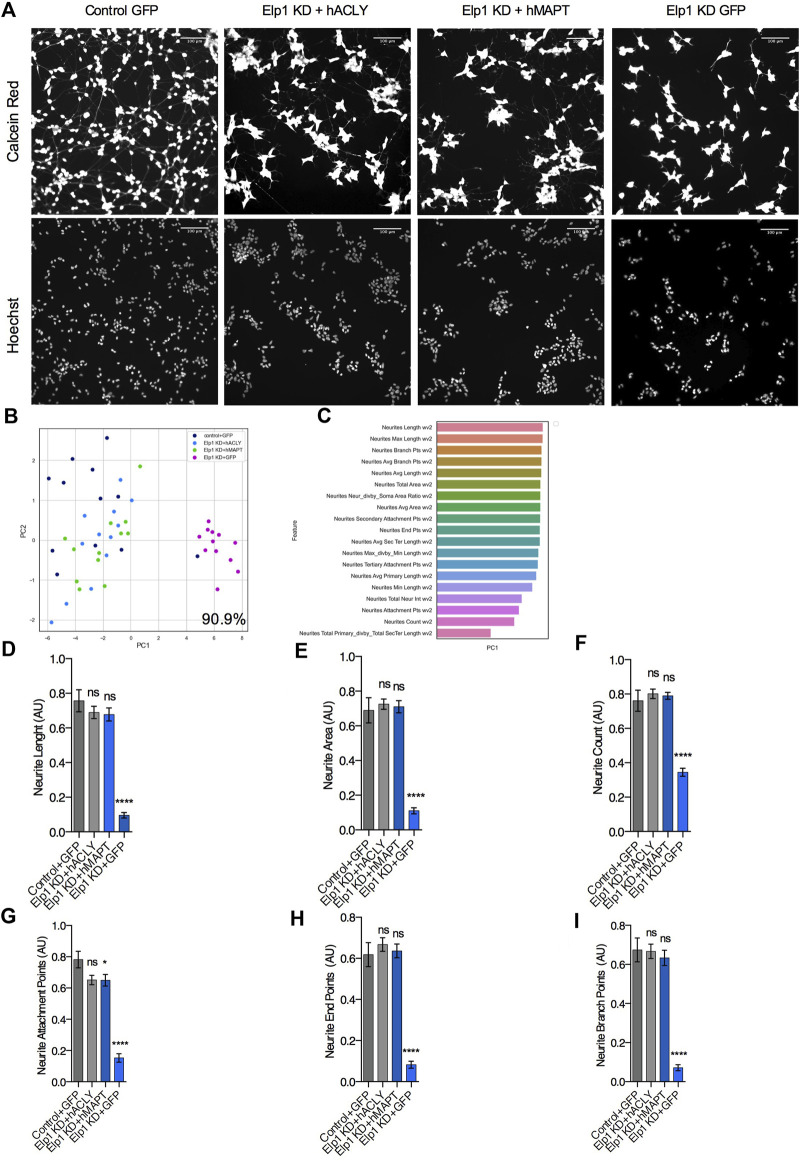
Expression of Acly or Tau rescues both Tau levels and neurites morphology defects in Elp1 KD cells. **(A)** Representative images of live fluorescence microscopy of differentiated SHSY5Y cells transfected with control GFP or MAPT or ACLY expressing plasmids stained with both Calcein AM (Red) and Hoechst (Blue); control (Control + GFP), Elp1 KD (Elp1KD + GFP), Elp1 KD expressing Tau (Elp1KD + hMAPT), Elp1 KD expressing acly (Elp1KD + hACLY) to visualize neurite morphology. Scale bar is 50 µm. **(B)** Two dimension principal component analysis (PCA) plot of stained cells based on morphological features. Symbols represent the control cells (dark blue); Elp1KD + hACLY cells (light blue), Elp1KD + GFP (pink), Elp1KD + hMAPT (green). each circle represent data extracted from 20 fields coming from one well, each field contain hundreds of cells. PCA Score 90.9% **(C)** Plot of features contribution to the separation in the PCA analysis **(D–I)** Quantification of the selected morphology features including Neurite Count **(D)**, Neurite Length **(E)**, Neurite Area **(F)**, Neurite Attachment Points **(G)**, Neurite End Points **(H)** and Neurite Branch Points **(I)**. All graphs show values of means ± SEM. Significance was determined by: e-j two-sided one-way analysis of variance (ANOVA), Specifically, [**(D)** *p* < 0.0001, F = 38.78; **(E)** *p* < 0.0001, F = 54.43; **(F)** *p* < 0.0001, F = 46.52; **(G)** *p* < 0.0001, F = 53.2; **(H)** *p* < 0.0001, F = 50.29; **(I)** *p* < 0.0001] In addition, the post hoc multiple comparisons, to analyze statistical difference of each condition compared to control for **(D–I)** by Dunnett test ****p < 0.0001. *n* = Number of wells (each well contains data from 20 fields, each field contains hundreds of cells): **(D–I)** Control + GFP *n* = 12; Elp1KD + GFP *n* = 12; Elp1KD + hACLY *n* = 12; Elp1KD + hMAPT *n* = 12.

Having established the molecular network linking Tau instability with the depletion of Elongator’s activity and its implication in neuritogenesis in neuroblastoma cells, we next investigated increasing Acly or Tau expression levels may rescue Elp1KD phenotype. Therefore, we simultaneously infected Elp1KD and control neuroblastoma cells with an adenovirus vector encoding for either Tau or Acly, 36 h prior their shorter RA-mediated differentiation to maximize the ectopic protein overexpression before neurite differentiation takes place. In order to investigate separately the Tau and Acly overexpression effects on neurite morphology, we compared the morphology data obtained from images of control neuroblastoma GFP and Elp1KD GFP infected cells, Elp1KD cells expressing human Tau and Elp1KD cells expressing human Acly (Figure 7). Figure 7A shows representative images of the neuroblastoma cell groups overexpressing the different proteins, GFP, Tau and Acly. Note that confluency in these experiments is lower due to viral infection affecting also the cell clumping phenotype in Elp1KD. As shown in Figure 7B, the two-dimensional principal component analysis applied to classify the difference in neurite morphology between GFP expressing control (in dark blue) and Elp1 KD (in pink), Elp1KD expressing Tau (hMAPT, in green) and Elp1KD expressing hACLY (in light blue) cells, resulted in 90.9% separation between the Elp1 groups overexpressing either Tau or Acly including the GFP neuroblastoma control group and the Elp1KD-GFP group. The selected neurite morphology features relevant for the PCA classification between the groups are shown in Figure 7C. Moreover, quantitative comparative analysis of the most relevant features between the groups are shown in Figures 7D–I where the overexpression effects of both Tau and Acly in Elp1 KD cells on each one of the morphological features in each of these graphs, is significantly different from the Elp1-KD-GFP group shown.

**FIGURE 8:**
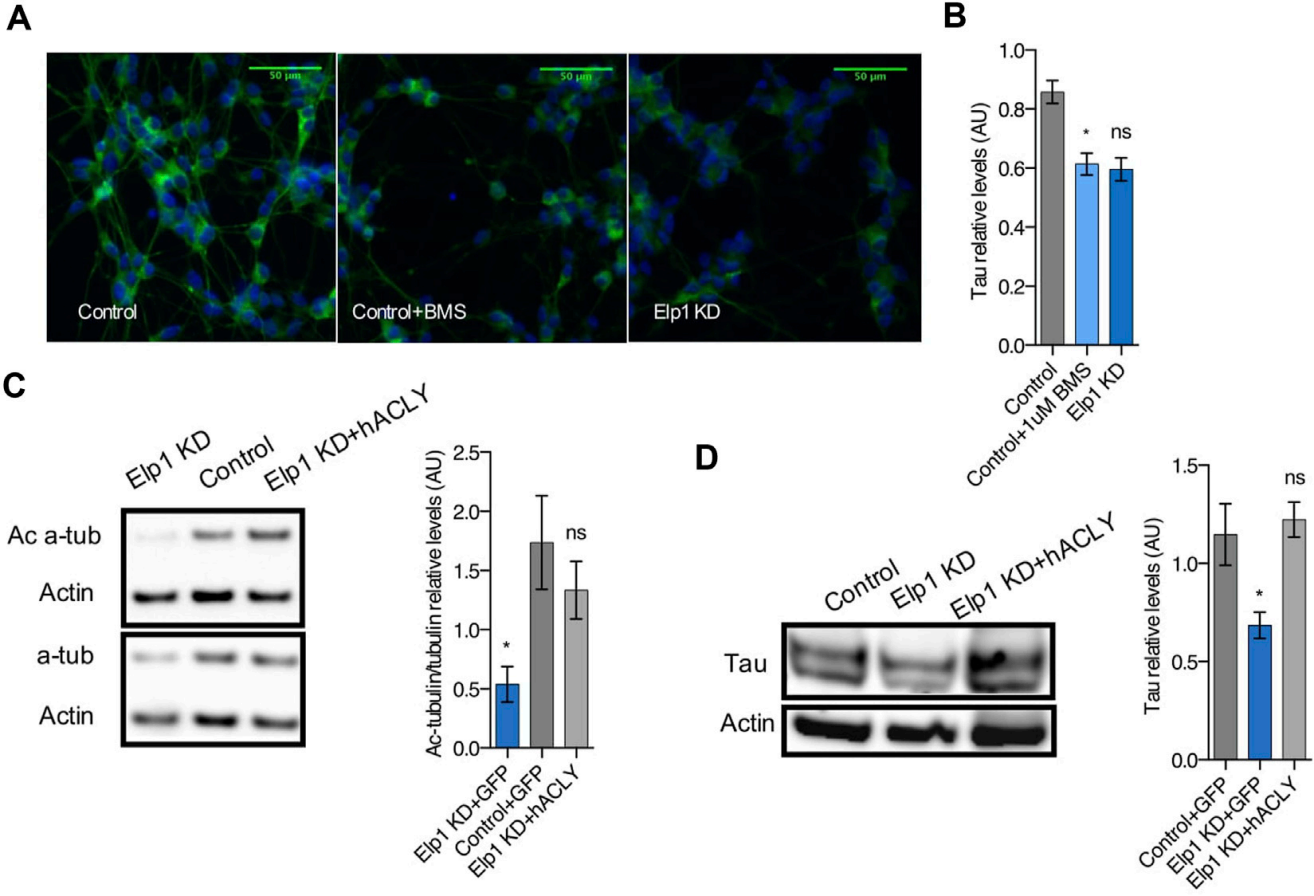
Inhibition of Acly interferes with Tau stability and Ac-α-tubulin levels establishing hierarchy involved in neurite morphology defects in Elp1 KD cells*.* **(A–B)** Immunolabeling and quantification of Tau levels in differentiated SHSY5Y extracts from Elp1KD cells and Control cells incubated with vehicle or BMS 303141 (BMS, 1 µM) for 72h. Scale bar is 50 µm. **(C–D)** Immunoblotting and quantification of Acetylated α-tubulin (Ac α-Tub), Total α-tubulin (t α-Tub) **(C)** and Tau **(D)** from differentiated SHSY5Y extracts from Control and Elp1 KD cells transfected with ACLY or GFP carrying constructs. All graphs show values of means ± SEM. Significance was determined by: **(B–D)** two-sided one-way analysis of variance (ANOVA), Specifically, [ **(B)** *p* = 0.0072, F = 6.112; **(C)** *p* = 0.0405, F = 4.494; **(D)** *p* = 0.0277, F = 5.487; ] In addition, the post hoc multiple comparisons, to analyze statistical difference of each condition compared to control for **(B–D)** by Dunnett test *p < 0.05. *n* = Number of wells (each well contains data from 20 fields, each field contains hundreds of cells): **(B)** Control *n* = 4; Control + BMS *n* = 7; Elp1KD *n* = 16; *n* = Number of experimental repeats: **(C)** Control + GFP *n* = 4; Elp1KD + GFP *n* = 4; Elp1KD + hACLY *n* = 5; **(D)** Control + GFP *n* = 4; Elp1KD + GFP *n* = 3; Elp1KD + hACLY *n* = 5.

To establish a hierarchy between Tau and Acly in the system, we tested whether Acly is required for the stability of Tau protein by specifically inhibiting Acly using BMS-303141 (BMS) (Li et al., 2007) in control differentiated neuroblastoma cells (Figure 8). This treatment reduced significantly the level of Tau protein compared to the Tau levels found in Elp1KD cells (Figures 8A,B) as judged by quantitative immunofluorescence analysis. This finding strongly suggests that Tau stability depends on Acly activity, establishing a regulatory relationship between the two in this system. Moreover, Acly overexpression in Elp1KD cells restores both the level of Tau and the level of ac α-tubulin (Figures 8C,D) confirming the dependence of Acly activity for Tau and MT integrity under Elongator deficiency background in these cells.

**FIGURE 9:**
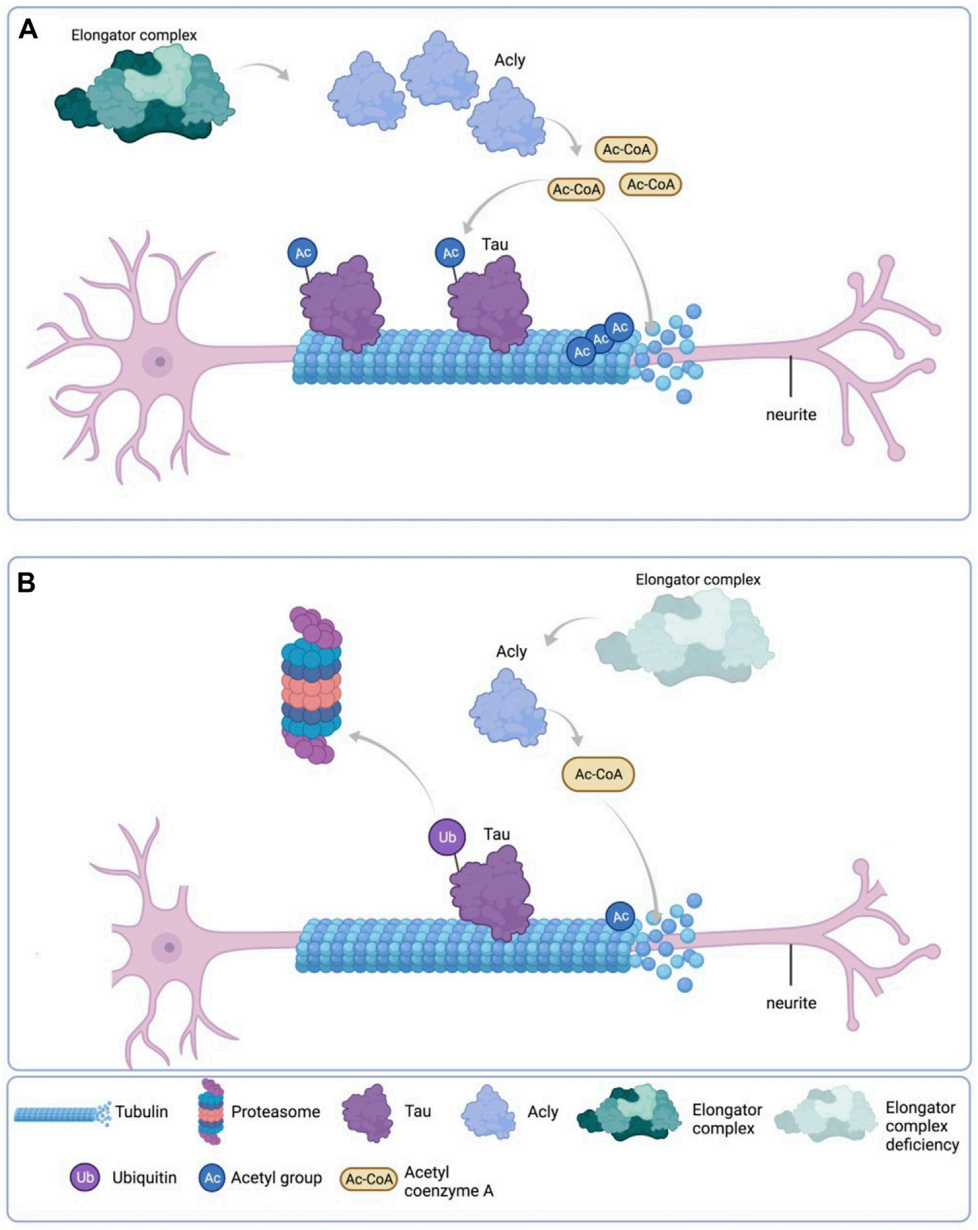
Schematic representation of the results. **(A)** Under normal Elongator complex activity, Acly’s enzymatic activity in the cytoplasm of neurons produces sufficient Ac-CoA for maintenance of acetylation levels of both Tau and MT *via* their respective acetyltransferases, contributing to normal neurite morphology. **(B)** Under Elongator complex deficiency (hElp1-KD), Acly’s enzymatic activity is compromised due to protein instability producing low Ac-CoA in the cytoplasm of neurons compromising acetylation levels required for maintenance of both Tau and MT causing polyubiquitylation and proteasomal degradation of Tau, contributing to MT instability and abnormal neurite morphology. Adapted from “Neuron Anatomy”, by BioRender.com (September 2020). Retrieved from https://app.biorender.com/biorender-templates/t-5f5b7e6139954000b2bde860-neuron-anatomy Copyright 2022 by BioRender.

Altogether, these results, as depicted in the cartoon in Figure 9, suggest that reduction of Acly expression causes reduction of MTs acetylation and Tau protein levels which directly affect the neurite morphology phenotype in Elp1KD neuroblastoma FD cells.

## Discussion

Here, we show that loss of Elongator activity impairs Tau stability *via* reduction of expression of Acly. This directly affects the neurite morphology, as exemplified in Elp1KD neuroblastoma cells. The neurite morphology defects observed in Elp1 KD are shared by Tau KD neuroblastoma cells, including features that relate to neurite network organization and neurite outgrowth that are correlated with the Tau expression levels in these cells. Evidence from different Tau knockout and knockdown models has shown that this protein is required for neurite outgrowth, which resulted in reduced neurite number and length, suppression of axon elongation and progressive degeneration of neurons (Liu, Lee and Jay, 1999; Hana N. Dawson et al., 2001; Ke et al., 2012; Bolkan and Kretzschmar, 2014). This may imply that both Elp1 and Tau deficiencies share a common pathway that is involved in the neurite phenotype in the FD cells. It has been shown that Tau plays significant role in the peripheral nervous system; it increases the structural stability and contributes to the high rate of axonal transport observed in peripheral axons (Tolkovsky and Brelstaff, 2018; Yi et al., 2019; Marquez et al., 2021) Peripheral neuropathy, like FD (Tourtellotte, 2016), results from neuronal dysfunction due in part to a loss of neurotrophic support (Sahenk, 2006), and since Tau plays an important role in stabilizing neuronal microtubules and regulating axonal transport, the malfunction of Tau may cause peripheral nerve dysfunction and degeneration (Marquez et al., 2021). The reduced levels of Tau observed upon Elongator depletion may contribute to FD peripheral nerve degeneration together with our previous findings of transport defects resulting from impaired α-tubulin acetylation (Noack, Leyk and Richter-Landsberg, 2014; Even et al., 2021). In addition, Tau malfunction has been associated with several neurodegenerative disorders, including frontotemporal dementia, Alzheimer, parkinsonism and other Tauopathies (Spillantini et al., 1998; Ludolph et al., 2009) triggering neurodegeneration. Although the deleterious effects of Tau pathology in those diseases are still highly debated, there is an assumption that Tauopathies are the consequence of loss of the ability of Tau to bind to and promote the assembly of microtubules (Lee and Leugers, 2012). Evidence of neurodegenerative diseases involving Tau downregulation are yet to be reported, but the connection between Tau misfunction and neurodegeneration is well described by others.

Moreover, we show that the reduced levels of Tau occur in other Elongator depleted models *in vitro* and *in vivo*. Therefore, we hypothesize that Elongator indirectly regulates Tau expression. Our results show in differentiated Elp1 KD neuroblastoma cells that Tau instability results from its hypoacetylation, which in turn increases its UPS degradation. This phenotype could be reversed by treatment with either or both Hdac6 and proteasomal degradation inhibitors treatment (Figure 4D, Figure 6A). It is already known that the hypoacetylation of Tau causes its reduced stability through UPS (Min et al., 2010). Therefore, an imbalance between acetylation and ubiquitylation at Tau lysine residues causes the dramatic reduction of Tau in Elp1 KD cells, which results from impaired acetylation due to reduced activity of Acly (see Figure 5, Figure 6) under the Elongator depletion background. This is part of a global and conserved mechanism by the Elongator regulating cellular acetylation processes through Acly which so far we have found that it is involved in α-tubulin acetylation, MT stabilization and neuronal transport and now Tau stability that is compromised in Elongator depleted models (Even et al., 2021).

Therefore, it is possible that under such hypoacetylation conditions increased proteasomal degradation of multiple proteins takes place in FD cells by default which might have a profound impact in neuronal function and survival. Ibrahim and others’ work on FD human olfactory ectomesenchymal stem cells (hOE-MSCs) supports this assumption (Hervé and Chérif, 2017) where Elp1 deficiency could induce proteasome alterations and that Elongator dysfunction in FD disturbs proteasome activity. Together with it, transcriptome and proteome analyses that have been done on DRG of mice in which Elp1 is conditionally ablated in the peripheral nervous system (PNS) suggest that the translational defects observed in Elongator loss are affecting downstream misregulation of numerous genes that function in ubiquitination, resulting in polyubiquitylation and proteasomal degradation of proteins (Goffena et al., 2018).

We propose that Elongator indirectly contributes to global cell levels of Ac-CoA *via* stabilization of Acly, which is regulating Tau stability *via* a fine balance between its lysine acetylation/ubiquitylation. This process is key to allowing proper neuritogenesis in differentiating neuroblastoma cells. Under Elongator deficiency in Elp1KD neuroblastoma cells, due to Acly instability, the reduced amount of available acetyl-CoA, that donates the acetate group needed for proteins acetylation, results in hypoacetylation of alpha-tubulin and Tau (which might affect also other protein targets) and their concomitant instability which has a direct profound impact in neurite morphology and vesicular transport as described previously (Even et al., 2021) (see Figure 9). Interestingly, overexpression of Tau and Acly recovers neurite morphology but apparently not the known cell-cell adhesion Contactin-1 dependent phenotype in Elp1 KD cells (Cohen-kupiec et al., 2010), as judged by the similar nuclei density and cell clusters observed in the images of the different groups (see Figure 7A). This suggests that the distinct Elp1 KD neuritogenesis Tau-dependent phenotype might be regulated separately from the Contactin-1 dependent one.

In conclusion, loss of Elongator activity might contribute to FD neuropathy by interfering with Acly expression, thereby MT-dependent transport and Tau levels. Altogether these results suggest that boosting Acly expression/activity should be investigated to implement future therapy for FD as well as to prevent the progression of other neurodegenerative disorders characterized by poor axonal transport, an impairment of tubulin acetylation or malfunction of Tau.

## Data Availability

The raw data supporting the conclusions of this article will be made available by the authors, without undue reservation.

## Supplementary material

Supplementary Figure 1 Elongator depletion leads to reduction in Tau protein expression **(A,B)** Western blotting to detect and quantify Tau, and ß-Actin in cortical extracts from newborn WT and Elp3 cKO mice. **(B)** Histogram of proportion of Tau expression to ß-Actin. **(C,D)** Western blotting to detect and quantify Tau, and ß-Actin in adult fly head extracts following neuronal (elav-driven) expression of wt and Elp3 KD. **(D)** Histogram of proportion of Tau expression to ß-Actin. All graphs show values of means ± SEM. Significance was determined by: b,d two-sided t test, Specifically, [**(B)** p = 0.0161; **(D)** p = 0.0216; **(B)** n = Number of mice: Control n = 4; Elp3 cKO n = 4; **(D)** n = Number of experimental repeats: Control n = 5; Elp3 KD n = 5.

Supplementary Figure 2 Tau is hypoacetylated, a result of impaired production of acetyl-CoA upon Elongator depletion. **(A)** Western blotting to detect and quantify acetylated alpha tubulin, and ß-Actin in differentiated SHSY5Y extracts from Control and Elp1 KD cells. Histogram of proportion of acetylated alpha tubulin expression to ß-Actin. **(B)** In vitro acetylation assay of non-acetylated MTs from HeLa cells incubated for 2 hours with extracts of differentiated SHSY5Y Control and Elp1 KD cells. All graphs show values of means ± SEM. Significance was determined by: b two-sided t test, Specifically, [p = 0.0106] **(D)** one-way analysis of variance (ANOVA), Specifically, [p < 0.0001, F = 1059]. n = Number of experimental repeats: **(B)** Control n = 5; Elp1 KD n = 5; **(D)** Control n = 6; WT n = 3 Elp1 KD n = 3.
