# Supplementary figures and images for: Elongator promotes neuritogenesis via regulation of tau stability through acly activity
Source: Front Cell Dev Biol. 2022 Oct 26;10:1015125. doi: 10.3389/fcell.2022.1015125 (PMC9644021; doi:10.3389/fcell.2022.1015125)

**A**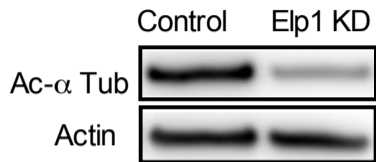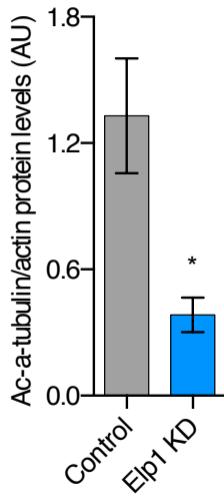**B**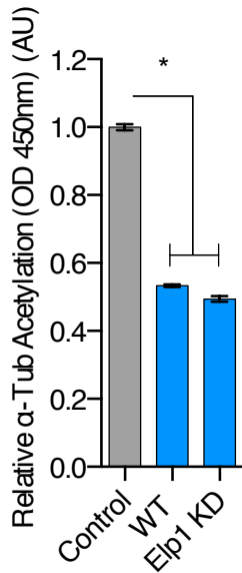

Supplement: Supplementary file 1 [file DataSheet2.PDF]

**A**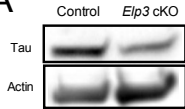**B**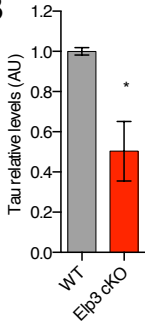**C**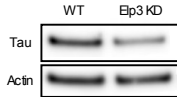**D**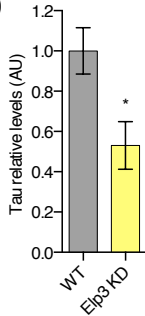

Supplement: Supplementary file 2 [file DataSheet1.PDF]
